# Translational Insights into Interferon Alpha’s Effects on Immunomolecular Dynamics in Philadelphia-Negative Myeloproliferative Neoplasms

**DOI:** 10.3390/cancers17142273

**Published:** 2025-07-08

**Authors:** Regina García-Delgado, Elena Luque-Lupiáñez, David Mora-Infante, Rodolfo Matías Ortiz-Flores, Borja Cidoncha-Morcillo, Julio Torres-González, Andrés Fontalba-Navas, Alejandro Escamilla-Sánchez

**Affiliations:** 1 UGC Hematología y Hemoterapia, Hospital Universitario Virgen de la Victoria, Servicio Andaluz de Salud, 29010 Malaga, Spain; regina.garcia.delgado.sspa@juntadeandalucia.es (R.G.-D.); elenaluquelupianez@gmail.com (E.L.-L.); borja.cidoncha.sspa@juntadeandalucia.es (B.C.-M.); 2 BE21-Hematología e Inmunoterapia, Instituto de Investigación Biomédica de Málaga y Plataforma en Nanomedicina (IBIMA Plataforma BIONAND), 29071 Malaga, Spain; jtorres.bbm@gmail.com (J.T.-G.); afontalba@uma.es (A.F.-N.); 3 Departamento de Fisiología Humana, Histología Humana, Anatomía Patológica y Educación Físico-Deportiva, Unidad Docente de Histología, Facultad de Medicina, Universidad de Malaga, 29071 Malaga, Spain; davidmorainfante@gmail.com; 4 Departamento de Salud Pública y Psiquiatría, Facultad de Medicina, Universidad de Malaga, 29071 Malaga, Spain; 5 Unidad de Salud Mental, Hospital de Antequera, Área Sanitaria Norte de Malaga, Servicio Andaluz de Salud, 29200 Malaga, Spain

**Keywords:** myeloproliferative neoplasms, interferon alpha, cytokines, gene expression, biomarkers, immunomodulation, PBMC

## Abstract

**Background/Objectives:** Interferon alpha (IFNα) remains a cornerstone in the management of Philadelphia-negative myeloproliferative neoplasms (Ph-neg MPNs), yet its immunomolecular impact over time is not fully elucidated. The aim of the study was to explore how IFNα therapy dynamically reshapes immune and gene profiles in Ph-neg MPNs and assess their potential as treatment-related biomarkers. **Methods:** This single-center, prospective, observational study included a translational substudy conducted within a previously established clinical cohort of 44 IFNα-treated patients, selecting a representative subset of 18 individuals stratified by treatment duration. Cytokine profiling (ELISA) and gene expression (RT-qPCR) analysis were performed using plasma and peripheral blood mononuclear cells (PBMCs), respectively. **Results:** Patients with prolonged exposure showed reduced pro-inflammatory cytokines and downregulation of inflammatory-signalling STAT1/STAT3 expression. In contrast, those with intermediate exposure exhibited transient TH2/regulatory cytokine peaks and upregulation of immunomodulatory genes such as CXCL10, SOCS3, and TNFAIP3. Spearman correlations revealed functional associations between cytokine and gene expression patterns including notable links such as STAT1–IL-13 and MYB–IL-13. **Conclusions:** These results describe a sequential immune reprogramming driven by IFNα, supporting the development of dynamic immunomolecular biomarkers of response in Ph-neg MPNs.

## Simple Summary

Patients with chronic blood cancers called myeloproliferative neoplasms (MPNs) can benefit from a therapy based on interferon alpha, which helps the immune system fight disease. However, we still do not fully understand how this treatment works at the molecular level, or how to predict who will respond best. In this study, we analyzed blood samples from patients treated with interferon alpha to investigate changes in immune signals and gene activity over time. We found a progressive decrease in inflammation-related molecules and changes in genes involved in immune regulation, cell survival, and blood cell production. These results suggest that interferon gradually reshapes the immune system and may help control the disease by reducing inflammation and promoting cell death in abnormal blood cells. Our findings may help identify new biomarkers to personalize treatment and monitor its effectiveness in future patients.

## 1. Introduction

Philadelphia-negative myeloproliferative neoplasms (Ph-neg MPNs), including polycythaemia vera (PV), essential thrombocythemia (ET), and primary myelofibrosis (PMF), are clonal disorders of the hematopoietic stem cell marked by chronic inflammation and abnormal haematopoiesis, driven by interactions between malignant clones and the bone marrow microenvironment [1]. Both innate and adaptive immune compartments contribute to disease evolution and immune evasion in these neoplasms [2]. Defective immune surveillance is associated with impaired antigen presentation, increased expression of checkpoint molecules (e.g., PD-1/PD-L1, CTLA-4), and expansion of immunosuppressive cell subsets, including regulatory T cells and myeloid-derived suppressor cells [3]. This immunosuppressive context facilitates clonal expansion and may underlie the limited success of immunotherapy in this patient population.

A key driver of this immunopathology is the cytokine milieu. Persistent overproduction of pro-inflammatory cytokines—such as interleukin-1β (IL-1β), interleukin-6 (IL-6), tumour necrosis factor-alpha (TNF-α), and interferon-gamma (IFN-γ)—promotes hematopoietic stem cell exhaustion, fibrotic progression, and disease advancement [4,5]. These inflammatory mediators reshape the bone marrow niche into a permissive environment for malignant haematopoiesis. Elevated cytokine levels are also detectable in pre-malignant conditions such as clonal haematopoiesis of indeterminate potential (CHIP) and myelodysplastic syndromes (MDS), emphasizing their broader relevance across myeloid malignancies. The efficacy of anti-cytokine therapies, including IL-6 inhibitors, in related hematologic diseases underscores the therapeutic potential of modulating inflammatory pathways in MPNs [5].

Philadelphia-negative MPNs are primarily driven by somatic mutations in JAK2, CALR, or MPL, which lead to constitutive activation of the JAK-STAT signalling pathway, resulting in cytokine hypersensitivity and uncontrolled myeloproliferation [1,4]. The current therapeutic landscape is dominated by JAK inhibitors such as ruxolitinib, which effectively reduce splenomegaly and systemic inflammation. Nevertheless, these agents can impair immune function and rarely induce durable molecular remissions or halt disease progression [4,6]. Immune checkpoint inhibitors have also yielded limited clinical benefit in this setting, and their use in a chronically inflamed bone marrow niche remains challenging due to the risk of exacerbating cytokine-driven pathology [4,7,8]. These limitations underscore the need for alternative or complementary strategies capable of restoring immune surveillance while suppressing malignant haematopoiesis.

Interferon alpha (IFNα) is a pleiotropic cytokine with established roles in immune modulation, hematopoietic regulation, and apoptosis. In MPNs, particularly those harbouring JAK2V617F mutations, IFNα has demonstrated efficacy in reducing allelic burden and achieving both hematologic and molecular responses [9,10]. Its therapeutic effects are mediated via activation of the JAK-STAT pathway and subsequent induction of interferon-stimulated genes (ISGs), which regulate cell cycle arrest, immune activation, and antiproliferative signalling. Pegylated IFNα formulations have enhanced pharmacokinetics and tolerability, facilitating long-term disease control in selected patients. Importantly, IFNα preferentially targets malignant stem and progenitor cells while preserving normal haematopoiesis and may slow or reverse fibrotic transformation [9]. However, prolonged IFNα exposure may also expand immunoregulatory subsets—such as CD56^+^ natural killer (NK) cells—highlighting the complex, context-dependent nature of its immunomodulatory activity [4,7].

Beyond its established effects on clonal burden, IFNα exerts broad immunomodulatory functions by altering cytokine secretion and transcriptional programs in immune cells. Preclinical studies have demonstrated that type I interferons enhance anti-tumour immunity by improving dendritic cell activation and promoting CD8^+^ T-cell responses. In murine models, loss of the IFNAR1 receptor impairs this axis, while IFNα restores immune competence via STAT1-dependent mechanisms [11]. Furthermore, IFNα induces transcriptional reprogramming of key immune regulators such as STAT1, SOCS3, and TNFAIP3, contributing to the resolution of inflammation and the restoration of immune homeostasis [12]. Cytokine studies confirm its dual-phase effect, as IFNα downregulates pro-inflammatory mediators (e.g., IL-6, TNF-α) while upregulating anti-inflammatory cytokines such as IL-10 [13].

Despite these advances, a critical knowledge gap remains: few longitudinal, integrated studies have assessed how IFNα modulates immunological parameters over time in real-world MPN patients. Most existing evidence is limited to short-term or cross-sectional observations, hindering our understanding of the temporal dynamics of cytokine production and gene expression during therapy. Given that IFNα may exert delayed but sustained effects on both clonal behaviour and immune function, elucidating the sequential immune reprogramming it induces is essential to guide biomarker discovery and personalized treatment approaches.

To address this gap, we conducted a translational study integrating cytokine profiling and gene expression analysis in peripheral blood mononuclear cells (PBMCs) from Ph-neg MPN patients undergoing IFNα therapy. Our aim was to characterize longitudinal immunological remodelling across different treatment durations. We hypothesized that IFNα elicits a time-dependent reconfiguration of cytokine networks and transcriptional pathways. By identifying coherent cytokine–gene shifts, our goal was to define candidate dynamic biomarkers predictive of treatment response, immune exhaustion, or therapeutic resistance in this immunologically complex disease.

## 2. Materials and Methods

### 2.1. Patients and Study Design

This study involved a retrospective cohort of 44 adult patients diagnosed with Philadelphia-negative myeloproliferative neoplasms (Ph-neg MPNs), all of whom received interferon alpha (IFNα) therapy at Hospital Universitario Virgen de la Victoria (Málaga, Spain). A representative molecular subset comprising 18 patients was selected—these were the individuals who remained on active therapy at the time of analysis, whereas the remainder had discontinued treatment due to adverse events, disease progression, or death. The cohort was stratified into three groups based on IFNα treatment duration: Group I, patients in the early phase of treatment (<6 months), to assess acute immune activation; Group II, patients in an intermediate stage (6–18 months), to explore immune adaptation and potential modulation; and Group III, patients in a late phase (>18 months), to investigate immune remodelling or exhaustion. Clinical data were retrieved through a structured review of institutional medical records, ensuring standardized collection of diagnostics, therapeutic, and laboratory parameters relevant to disease characterization and IFNα exposure.

### 2.2. Plasma Cytokine Profiling

Peripheral blood samples were collected in EDTA tubes during routine outpatient visits and processed within two hours of collection. Plasma was separated by centrifugation and stored at −80 °C until analysis. Cytokine concentrations were quantified using a multiplex bead-based immunoassay (Luminex^®^ platform; Luminex Corporation, Austin, TX, USA), following the manufacturer’s instructions. The following cytokines were measured: IL-1α, IL-1β, IL-6, IL-8, MCP-1, IFN-γ, TNF-α, IL-4, IL-10, and IL-13. Results are reported as mean concentrations in picograms per millilitre (pg/mL).

### 2.3. RT-qPCR Primer Design and Validation

RT-qPCR assays were conducted by AnyGenes SAS (Paris, France), a certified molecular diagnostics company specializing in targeted gene expression profiling. RT-qPCR data were analysed using CFX Manager™ Software version 3.1 (Bio-Rad, Hercules, CA, USA). Primer sequences were designed using NCBI Primer-BLAST, based on RefSeq transcripts, targeting the same exonic regions and amplicon sizes as defined in their pre-validated custom panel. Each primer pair was selected to ensure single-product amplification and high specificity. Although the proprietary nature of the original primers precludes disclosure, AnyGenes provided the corresponding in silico sequences, faithfully replicating the analytical targets employed experimentally. All primer pairs underwent internal quality control by the supplier, including melting curve analysis, single-product verification, and amplification efficiency testing (90–110%). This external validation adds methodological rigor and ensures the reproducibility of our gene expression data. The complete list of primer sequences, amplicon sizes, and genomic annotations is provided in Supplementary Table S1.

### 2.4. Gene Expression Analysis in Peripheral Blood Mononuclear Cells (PBMCs)

Peripheral blood mononuclear cells (PBMCs) were isolated from whole blood using Ficoll-Paque™ density-gradient centrifugation. Total RNA was extracted using a silica-column-based protocol (RNeasy Mini Kit, Qiagen, Singapore), and RNA integrity was assessed by spectrophotometry and agarose gel electrophoresis. Complementary DNA (cDNA) was synthesised using a reverse transcription kit, following the manufacturer’s instructions. Quantitative real-time PCR (qPCR) was performed using SYBR Green chemistry on a StepOnePlus™ thermocycler (Applied Biosystems, Waltham, MA, USA). Gene expression was assessed for the following transcripts: STAT1, STAT3, SOCS1, SOCS3, CXCL10, TNFAIP3, BAX, BCL2, GATA1, and MYB. Expression levels were normalized to HPRT1, a reference housekeeping gene with stable expression in PBMCs under IFNα stimulation. Relative expression was calculated using the 2^−ΔCq^ method and expressed as relative units (×10,000). The gene panel was selected based on functional relevance to IFNα-related immunomodulation and MPN biology. It encompasses markers of inflammatory signalling (e.g., CXCL10), immune regulation (TNFAIP3, SOCS1, SOCS3), apoptosis (BAX, BCL2), hematopoietic regulation (MYB, GATA1), and canonical IFNα-JAK-STAT signalling (STAT1, STAT3).

### 2.5. Correlation Analysis Between Gene Expression and Cytokines

To explore the functional relationships between transcriptional and immune signatures, Spearman correlation coefficients (ρ) were calculated between gene expression (2^−ΔCq^ × 10,000) and the corresponding plasma cytokine concentrations. This analysis was performed across the entire 18-patient molecular cohort, independent of treatment group stratification, to maximize statistical power and reveal global immunomolecular associations. Correlation patterns were visualized using a two-dimensional heatmap with color-coded ρ values and scatter plots with regression lines and 95% confidence intervals.

### 2.6. Statistical Analysis

Descriptive statistics were used to summarize clinical and molecular variables. Comparisons between treatment duration groups were performed using one-way ANOVA or Kruskal–Wallis tests, followed by Tukey’s or Dunn’s post hoc analysis, where appropriate. Correlations were assessed using Spearman rank correlation. A *p*-value < 0.05 was considered statistically significant. Data analysis and visualization were carried out using Python 3.10.6 (Pandas, SciPy, Seaborn) and GraphPad Prism version 9.0.

### 2.7. Ethical Statement

The study was approved by the institutional ethics committee under protocol SICEIA-2025-000113 (POLIVERA-INF project) and was conducted in accordance with the Declaration of Helsinki. The study included both retrospective and prospective components, focusing on the identification of predictive biomarkers in IFNα-treated Ph-neg MPNs. Blood samples were obtained during routine clinical care and processed under standardized biobanking conditions at the certified IBIMA-HUVV Biobank. All sample collection, handling, and storage followed institutional standard operating procedures (SOPs) to ensure nucleic acid integrity and assay reproducibility. All patients received detailed written information about the study and provided signed informed consent before participation, in accordance with institutional and international ethical guidelines.

## 3. Results

### 3.1. Baseline Characteristics of the Cohort

The study included 44 patients diagnosed with Ph-neg MPNs, including PV, ET, and PMF. All patients received treatment with interferon alpha (IFNα), with a median exposure duration of 8.2 months (range: 1–36 months). The detailed baseline characteristics are summarized in Table 1.

Clinical features observed in the cohort included pruritus (40.9%), splenomegaly (25%), and a prior history of thrombosis (13.6%). These variables were included in exploratory analyses to assess potential associations with immunological and molecular parameters. The overall clinical response rate to IFNα was 50%. As shown in Figure S1, no statistically significant association was found between the frequency of memory/activated CD8^+^ T cells and the duration of IFNα exposure (Figure S1A). Although viral monitoring was not predefined as a study endpoint, low-level viral reactivations were detected in a minority of patients. These episodes were clinically asymptomatic and did not require therapeutic intervention (Figure S1B).

### 3.2. Plasma Cytokine Profiling Across Treatment Time Points

To investigate the immunological effects of IFNα treatment, a subgroup of 18 patients underwent targeted molecular and cytokine analysis. This subgroup consisted of patients who were exposed to IFNα at different stages. Six patients were recruited at the beginning of their treatment (Group I), while the remaining patients were included retrospectively during routine clinical visits. Blood samples were collected from these patients at various points during their follow-up, with five patients in Group II and eight patients in Group III. The results showed statistically significant differences in several key immune mediators among the different treatment groups (Kruskal–Wallis test, Table S2).

**Figure 1:**
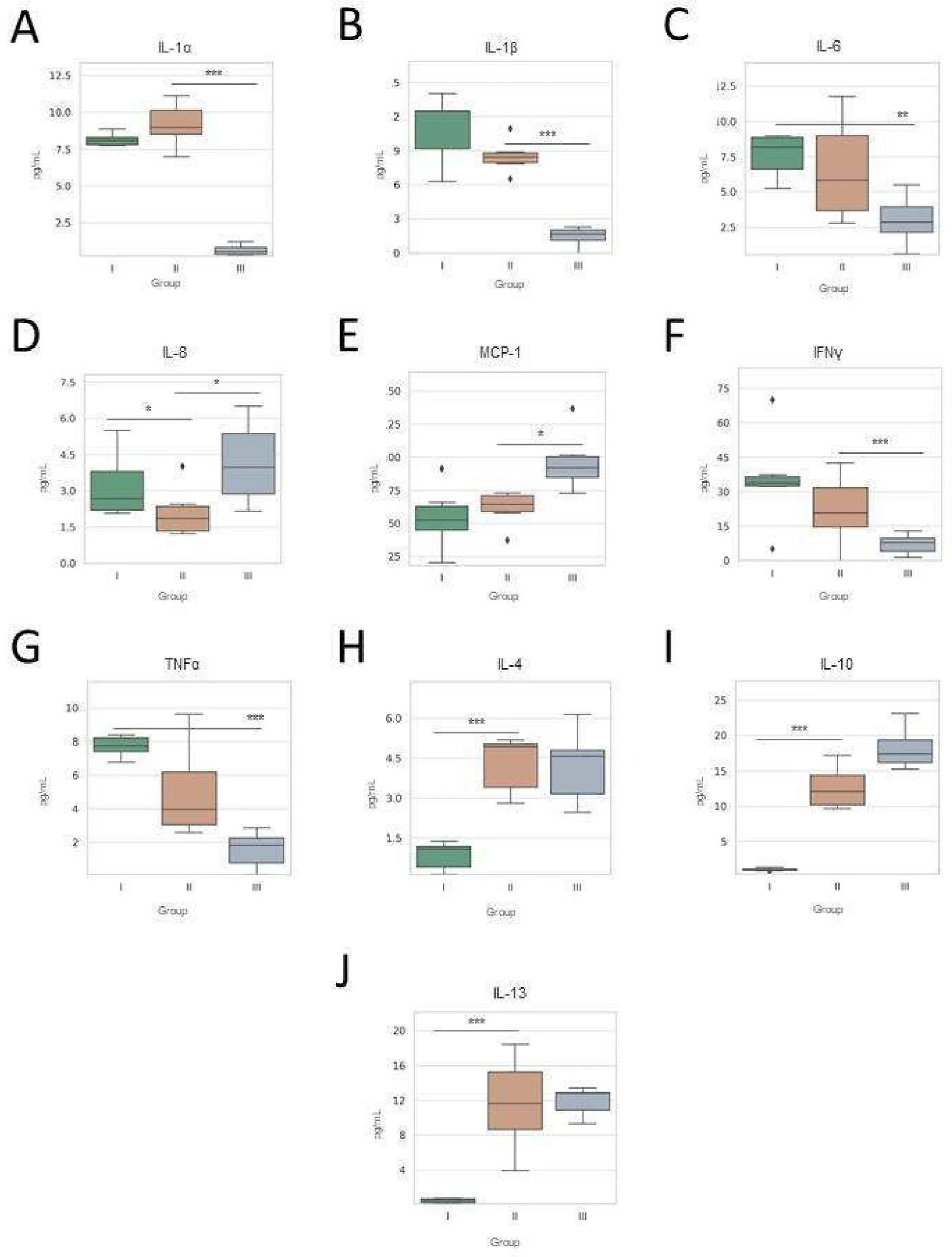
Plasma cytokine dynamics stratified by IFNα exposure group. Boxplots show plasma levels (pg/mL) of 10 cytokines measured in patients categorized by IFNα treatment duration: Group I (early exposure), Group II (intermediate), and Group III (prolonged). (**A**) IL-1α. (**B**) IL-1β. (**C**) IL-6. (**D**) IL-8. (**E**) MCP-1. (**F**) IFNγ. (**G**) TNFα. (**H**) IL-4. (**I**) IL-10. (**J**) IL-13. Asterisks indicate levels of statistical significance (*p* < 0.05: *, *p* < 0.01: **, *p* < 0.001: ***).

The levels of pro-inflammatory cytokines, such as IL-1β, TNFα, and IL-6, showed a significant decrease from Group I to Group III (*p* < 0.01), consistent with the progressive immunomodulatory effect of IFNα. IL-10 levels increased sharply from Group I to Group II and remained elevated in Group III, while IFNγ showed a parallel significant decline (*p* < 0.001), supporting an early immune modulation driven by IFNα. Furthermore, significant changes were observed in IL-1α, IL-4, and IL-13, reflecting broader shifts in pro- and anti-inflammatory signalling pathways. These findings are illustrated in Figure 1. The observed cytokine trajectories support the hypothesis of gradual immunological remodelling during IFNα therapy, which may contribute to clinical response and modulation of the disease phenotype.

### 3.3. Gene Expression Profiles Across IFNα Exposure Groups

To explore the transcriptional effects of IFNα exposure, gene expression was quantified in PBMCs from patients in all three treatment groups. The expression of ten selected genes relevant to inflammation, apoptosis, haematopoietic transcription, and interferon signalling pathways were quantified in PBMCs. Several genes showed statistically significant changes across the treatment duration groups (Kruskal–Wallis test). These results are summarized in Table S3.

**Figure 2:**
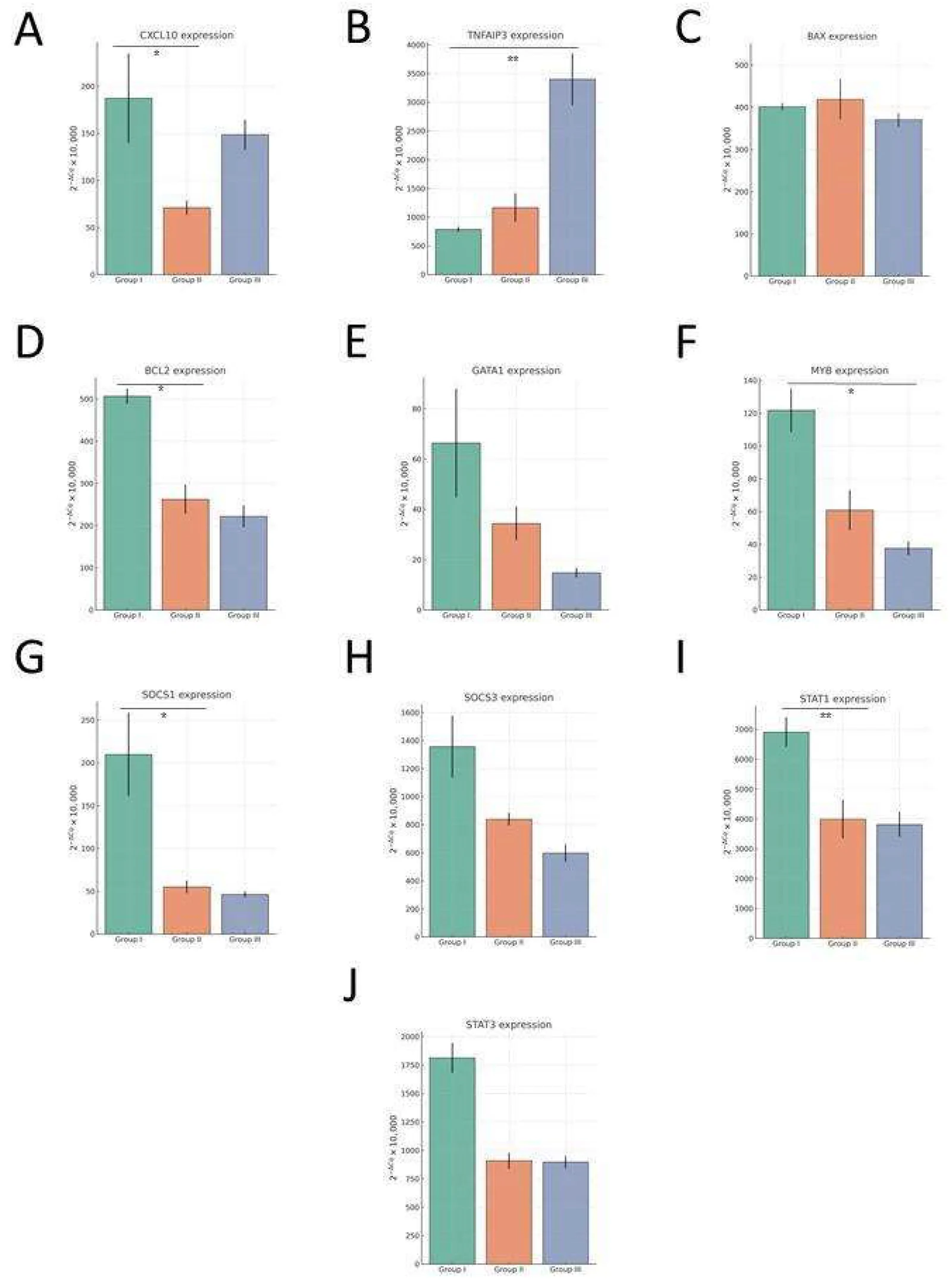
Gene expression dynamics of PBMCs under IFNα exposure in three patient groups. Bars represent mean ± SEM values of gene expression (2^−ΔCq^ × 10,000). Groups correspond to increasing durations of IFNα treatment (Group I: early; Group II: intermediate; Group III: prolonged). (**A**–**J**) Expression levels of selected genes, grouped by functional categories: (**A**,**B**) Interferon-response and chemokine genes: CXCL10 (**A**), TNFAIP3 (**B**); (**C**,**D**) Apoptosis regulators: BAX (**C**), BCL2 (**D**); (**E**,**F**) Hematopoietic transcription factors: GATA1 (**E**), MYB (**F**); (**G**–**J**) JAK/STAT pathway components: SOCS1 (**G**), SOCS3 (**H**), STAT1 (**I**), STAT3 (**J**). Asterisks indicate statistical significance between groups (*p* < 0.05: *, *p* < 0.01: **).

Among interferon-responsive genes, CXCL10 expression peaked in Group II patients and declined in Group III (*p* = 0.0261, Figure 2A), while TNFAIP3 levels showed a consistent and significant increase across the three groups (*p* = 0.0022, Figure 2B). BAX remained relatively stable (*p* = 0.32, Figure 2C) whereas BCL2 expression decreased significantly from Group I to III (*p* = 0.0148, Figure 2D). Regarding haematopoietic transcription factors, both GATA1 (*p* = 0.0521, Figure 2E) and MYB (*p* = 0.0132, Figure 2F) expression levels decreased progressively in Group III; SOCS1, a negative regulator of STAT signalling, was significantly downregulated, but SOCS3 (another negative regulator of STAT signalling) showed a non-significant downward trend (*p* = 0.0139, Figure 2G, and *p* = 0.2688, Figure 2H, respectively). STAT1 expression decreased significantly over time (*p* = 0.0066, Figure 2I). STAT3 showed a non-significant downward trend (*p* = 0.7856, Figure 2J).

### 3.4. Correlations Between Gene Expression and Cytokine Profile

Spearman correlation analysis of the 18-patient molecular cohort identified nominal associations between STAT1 expression and IL-13 (ρ = 0.819; *p* = 0.000033), STAT3 expression and IL-6 (ρ = 0.783; *p* = 0.000122), STAT3 expression and TNFα (ρ = 0.667; *p* = 0.00250), STAT3 expression and IL-10 (ρ = 0.656; *p* = 0.00310), MYB expression and IL-13 (ρ = −0.511; *p* = 0.0302), and BCL2 expression and TNFα (ρ = 0.504; *p* = 0.0328). After Benjamini–Hochberg correction across the complete set of 35 gene–cytokine correlations, STAT1–IL-13 (q = 0.00115), STAT3–IL-6 (q = 0.00214), STAT3–TNFα (q = 0.0272), and STAT3–IL-10 (q = 0.0272) remained statistically significant at FDR < 0.05. MYB–IL-13 and BCL2–TNFα did not remain significant after multiplicity correction (both q = 0.191). These findings are exploratory and require independent validation.

## 4. Discussion

IFN-α involves multiple mechanisms to fight cancer, including promoting cell death, slowing tumour growth, and modifying the immune system. Our study offers a comprehensive molecular overview of immunological remodelling in Ph-neg MPNs under IFN-α therapy by integrating cytokine profiling and gene expression data from a representative patient subset. Using a stratified design based on treatment duration, we identified progressive and coordinated alterations in pro-inflammatory cytokines, apoptotic markers, and haematopoietic transcriptional programmes. These results align with the existing literature, which emphasises that the immunological effects of IFN-α are not static but evolve in response to treatment duration and disease context [6].

Despite the limited sample size, the selected cohort captures the clinical and molecular heterogeneity of IFN-α–treated Ph-neg MPNs. Similar sample sizes have been employed in previous biomarker investigations in MPNs [14], particularly those employing stratified longitudinal analyses and integrated immunomolecular endpoints. As such, the current dataset provides a robust foundation for hypothesis generation regarding IFN-α–induced immunological reprogramming. These findings contribute to characterising the immunobiological trajectory associated with IFN-α exposure and lend support to the implementation of dynamic molecular monitoring in chronic neoplastic conditions [15]. One of the most consistent observations in our study was the progressive decline in pro-inflammatory cytokines (IL-1α, IL-1β, IL-6, IL-8, TNFα, and IFNγ), alongside Th2-associated cytokines (IL-13 and IL-4), in patients with extended IFN-α exposure. This dual decline supports a biphasic immunomodulatory model in which IFN-α induces immune activation and promotes an immunological silencing phase, potentially via homeostatic feedback or exhaustion mechanisms [16,17]. In the context of MPNs—where chronic inflammation perpetuates clonal expansion and fibrosis—this evolving cytokine profile is consistent with the recognised therapeutic actions of IFN-α. Specifically, the observed decrease in IL-13 and IL-4, both implicated in TGF-β-driven fibrosis and megakaryopoiesis, suggests that IFN-α may attenuate fibrotic progression [18]. Interestingly, despite the overall anti-inflammatory trend observed with longer treatment exposure, the levels of IL-8 and MCP-1 remained relatively stable across groups. This finding may reflect the distinct regulation of these chemokines, which are often less responsive to interferon signalling. For instance, IL-8 is predominantly active in neutrophils and monocytes—cell types that may exhibit reduced sensitivity to IFN-α—and its expression appears more dependent on localised microenvironmental cues than on systemic immune modulation [19]. Similarly, MCP-1 plays a critical role in monocyte recruitment and stromal activation, pathways that may become more pronounced during fibrotic progression. Prior research has associated elevated levels of IL-8 and MCP-1 with advanced MPN phenotypes and the development of marrow fibrosis [20,21]. Therefore, their stability in our cohort may reflect a relatively early fibrotic stage and reinforce their potential utility as biomarkers of fibrotic transformation. Altogether, these data underscore the value of longitudinal cytokine profiling as a surrogate marker of treatment duration and effectiveness and highlight the importance of dynamic immune monitoring in optimising therapeutic strategies.

Gene expression profiling in PBMCs revealed sequential modulation of interferon-responsive genes such as CXCL10 and TNFAIP3 (Figure 2A). CXCL10, a potent chemoattractant for activated T cells and NK cells, is classically induced by IFN-γ and type I interferons and is typically associated with Th1 polarisation. However, its role under chronic interferon exposure remains context-dependent. In our cohort, CXCL10 expression peaked in patients receiving IFNα for 3–6 months, suggesting an intermediate-phase immune activation that may represent a transient window of heightened IFNα responsiveness [18]. This temporary peak could reflect a therapeutic “sweet spot” where IFNα-induced immunostimulation is at its most effective before counter-regulatory mechanisms prevail. In contrast, TNFAIP3—a ubiquitin-modifying enzyme that acts as a negative regulator of NF-κB and TNF signalling—showed a gradual and sustained increase in expression over time [9]. This trajectory suggests that prolonged IFNα exposure promotes the upregulation of anti-inflammatory circuits aimed at maintaining immune homeostasis. TNFAIP3 has previously been implicated in the resolution of chronic inflammation and prevention of autoimmunity. Its induction in our setting likely reflects a protective adaptation that mitigates excessive immune stimulation associated with extended IFNα therapy. These opposing patterns of CXCL10 and TNFAIP3 expression mirror the biphasic cytokine responses described earlier, reinforcing the concept of phased immune adaptation: early immune activation is followed by a transcriptional silencing of inflammatory pathways. This dynamic shift is in line with clinical observations of reduced toxicity and improved tolerability in patients undergoing long-term IFNα therapy. The analysis of apoptosis-related gene expression revealed a progressive decline in BCL2 levels with prolonged IFNα exposure, while BAX expression remained largely unchanged (Figure 2B), resulting in a modest shift towards a pro-apoptotic profile. This finding aligns with the established anti-apoptotic role of BCL2 in the mitochondrial pathway [22] and prior evidence that IFNα can induce apoptosis in malignant progenitor cells, contributing to molecular remissions in MPNs [10,23]. The BAX–IL-10 association was inverse but did not reach nominal or FDR-adjusted statistical significance (ρ = −0.412; *p* = 0.0893; q = 0.3905). Accordingly, no mechanistic inference is made from this association, and the BAX/BCL2 expression findings are interpreted independently of this exploratory cytokine correlation [24].

The progressive downregulation of GATA1 and MYB (Figure 2C), two master regulators of erythroid and megakaryocytic differentiation, suggests a subtle yet consistent suppression of haematopoietic lineage commitment during IFNα therapy. While these transcription factors are indispensable for physiological haematopoiesis, they also play a crucial role in maintaining clonal progenitor expansion in Philadelphia-negative myeloproliferative neoplasms. In particular, MYB has been identified as a key effector of JAK2V617F-driven proliferation and is frequently upregulated in haematological malignancies characterised by aberrant stem or progenitor activity [25]. Our findings point towards transcriptional silencing of progenitor-supportive pathways, potentially reflecting the selective pressure imposed by IFNα on malignant haematopoietic clones. MYB–IL-13 showed a nominal inverse association (ρ = −0.511; *p* = 0.0302) that did not remain significant after BH-FDR correction (q = 0.1913). In contrast, STAT1–IL-13 showed a positive association (ρ = 0.819; *p* < 0.001; q = 0.00115) that remained significant after BH-FDR correction. Given the cross-sectional design, these associations are considered exploratory and should not be interpreted as evidence of causal or within-patient temporal relationships [26]. Although we did not directly assess progenitor frequencies or clonal architecture, the concerted suppression of MYB, GATA1, BCL2, and STAT1 in patients receiving prolonged IFNα therapy—together with the accompanying cytokine shifts—provides indirect evidence that IFNα may exert its therapeutic effects by targeting early disease-initiating cells. This mechanism could underlie the capacity of IFNα to induce molecular remissions. Collectively, our data support a model in which IFNα not only modulates the immune milieu but also progressively reshapes the transcriptional landscape of haematopoietic progenitors, potentially resulting in the exhaustion or silencing of malignant clones.

**Figure 3:**
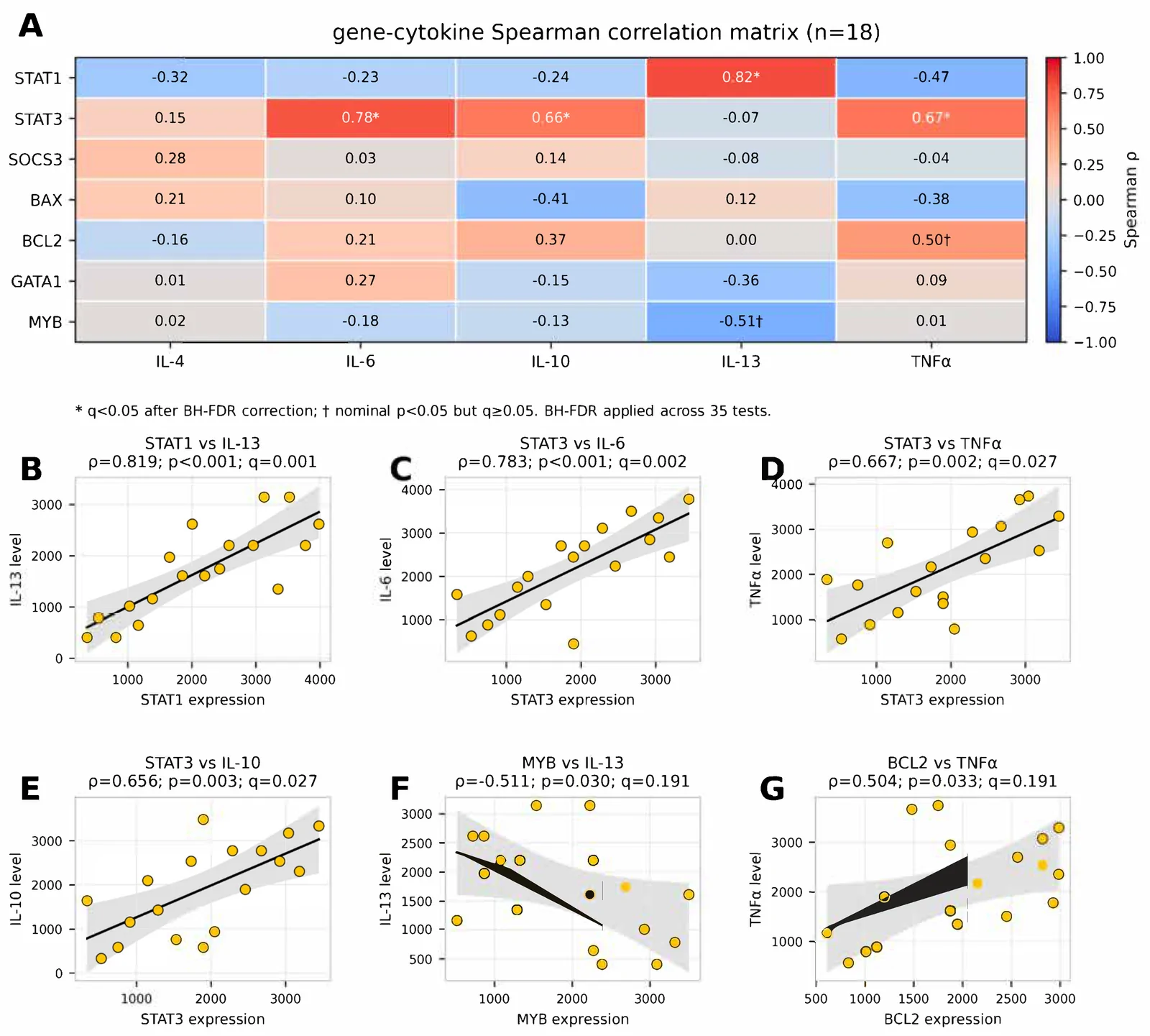
Gene–cytokine correlations in the IFNα-treated molecular cohort. (**A**) shows all predefined 7 × 5 Spearman correlations recalculated from patient-level data (*n* = 18). (**B**–**G**) show FDR-significant associations and nominal-only exploratory associations included in the corrected response. In panels (**B**–**G**), yellow circles represent individual patient observations, the solid black line represents the fitted linear regression line for visualization, and the grey shaded area denotes the 95% confidence interval.

Components of the JAK–STAT pathway exhibited a distinctive pattern of modulation under IFNα exposure. STAT1 expression declined significantly over time, whereas STAT3 levels remained stable (Figure 2D), suggesting an attenuation of pro-inflammatory signalling with preservation of survival-associated pathways. These divergent expression trajectories may reflect their distinct immunobiological functions during IFNα therapy. STAT1 activation is a hallmark of interferon signalling, driving the transcription of interferon-stimulated genes (ISGs), promoting apoptosis, and mediating clonal suppression of malignant progenitors [27]. However, sustained or excessive STAT1 activation has been associated with immune exhaustion, which could be counterbalanced by its progressive downregulation over time. In contrast, STAT3 supports anti-inflammatory and immune-regulatory processes. Its relative stability in response to IFNα exposure may serve to buffer excessive inflammation while preserving immune competence [28]. Previous studies in MPN patients have demonstrated that low baseline STAT1 expression with a high fold induction upon IFNα exposure correlates with molecular response, while unchecked STAT3 activity is implicated in immune escape in other malignancies [27]. Together, the modulation of these transcription factors suggests a transition from an initial immune activation phase to a state of regulated immune homeostasis, which may underpin the durability of IFNα responses in selected patients. Such immunological adaptation has been reported in models of sustained interferon signalling and may involve receptor internalisation, SOCS-independent regulatory mechanisms, or epigenetic modifications limiting access to ISG promoters (Figure 3). Among the predefined gene–cytokine correlations, STAT1–IL-13, STAT3–IL-6, STAT3–TNFα, and STAT3–IL-10 remained statistically significant after BH-FDR correction. MYB–IL-13 and BCL2–TNFα were nominal only, whereas STAT3–IL-4, SOCS3–IL-6, GATA1–IL-10, and BAX–IL-10 were non-significant. The interpretation has therefore been narrowed to avoid mechanistic conclusions based on associations that were not supported after multiplicity correction [29,30,31,32,33].

Key transcripts such as TNFAIP3, CXCL10, and STAT1 emerged as potential indicators of immune adaptation during prolonged IFNα exposure. These temporal immune shifts may hold significant translational relevance. Elevated CXCL10 and TNFAIP3 levels during intermediate phases of treatment may reflect an effective immunological activation window associated with clinical benefit [34]. In contrast, the progressive decline in STAT1, IL-13, and other pro-inflammatory mediators in patients treated long-term could indicate immune exhaustion or the onset of a therapeutic plateau [27]. Although this study was not powered to stratify patients by clinical response or toxicity, previous reports have demonstrated that low baseline STAT1 expression with high IFNα-induced fold change correlates with molecular response [27], whereas high baseline CXCL10 or IFN-γ levels may be associated with poor response or increased risk of immune-related toxicity [35]. In addition, dynamic changes in IL-10 may influence treatment tolerability, and the upregulation of TNFAIP3 may reflect a beneficial resolution of inflammation [34]. These observations support the concept that dynamic immunomolecular signatures could inform therapeutic decision-making in MPNs, and highlight the need for validation in larger, prospectively monitored patient cohorts. Although the current cohort was not stratified according to haematologic or molecular response, approximately half of the patients achieved remission, reinforcing the potential translational value of the identified molecular markers [10].

This study presents certain limitations, notably the restricted size of the molecular cohort (*n* = 18). Although patient selection was based on sample quality and was balanced across treatment duration groups, the small cohort size may limit the statistical power and generalisability of the findings. Nonetheless, the data generated here constitute a valuable starting point that may serve as a foundation—or even a disruptor—for future investigations. Similar cohort sizes have been employed in exploratory transcriptomic studies of rare diseases [36,37,38,39], yet validation in larger, multicentre, prospectively designed cohorts will be essential to confirm the reproducibility and clinical significance of these immunomolecular signatures. In addition, the observational nature of this study precludes causal inferences between treatment exposure and clinical outcomes. The absence of stratification by clinical response further limits the ability to detect differential molecular effects across patient subgroups. Despite these constraints, the patterns identified align with prior evidence of durable responses to pegylated IFNα and lend further support to the hypothesis that IFNα induces a time-dependent transition from inflammatory activation to regulatory and apoptotic reprogramming. This progressive immunological remodelling may underpin clonal suppression and sustained disease control. Importantly, the internal coherence observed between cytokine and gene expression profiles across treatment duration groups provides a solid basis for future biomarker development. Stratified, prospective clinical trials will be necessary to establish the predictive value of these molecular markers and to assess their integration with clinical endpoints. Our findings reinforce the utility of molecular phenotyping in chronic myeloid neoplasms and highlight the multifaceted role of IFNα in remodelling the immune landscape. Mechanistically, IFNα exerts its effects through sequential activation of the JAK–STAT, interferon regulatory factor (IRF), and NF-κB pathways. Upon engagement of the IFNAR receptor, STAT1 and STAT2 are phosphorylated, driving the transcription of interferon-stimulated genes (ISGs) such as CXCL10 and IRF7, which recruit T cells and natural killer (NK) cells [40]. This initiates an early pro-inflammatory phase, exemplified in our cohort by transient elevations of CXCL10, IL-6, TNFα, and IFNγ—consistent with Th1 polarisation and innate immune activation. Subsequently, increased expression of TNFAIP3 (A20) serves to inhibit NF-κB signalling and promote inflammatory resolution [41]. The concurrent rise in IL-10 and IL-13 supports a regulatory phase that may limit toxicity and promote immune tolerance [42]. This biphasic immunological sequence mirrors the clinical effects of IFNα, wherein early immune stimulation is followed by transcriptional dampening and homeostasis—potentially preventing immune exhaustion and facilitating long-term therapeutic benefit. Beyond generating hypotheses, this work outlines a conceptual framework for dynamic immune monitoring and precision-guided interferon therapy, reinforcing the rationale for integrating interferon-based strategies into personalized treatment algorithms for selected MPN patients.

## 5. Conclusions

This study provides novel insights into the immunomolecular characterisation of the mechanisms underlying interferon alpha (IFNα) therapy in Philadelphia-negative myeloproliferative neoplasms (Ph-neg MPNs). Through the integrated analysis of gene expression and plasma cytokine profiles, we identified a dynamic and coordinated immunological remodelling that evolves over the course of treatment. Collectively, these findings underscore the potential of dynamic molecular profiling to capture the shifting immune landscape in MPNs and to identify biomarkers that may guide treatment duration, intensity, or discontinuation. Although exploratory in nature, this study establishes a foundation for the prospective validation of transcriptional and cytokine markers—such as TNFAIP3, CXCL10, and STAT1—as components of precision-guided interferon therapy in chronic myeloid neoplasms. While the limited size of the molecular cohort remains a constraint, the results presented herein offer a compelling basis for further investigation. Larger, prospective studies will be necessary to confirm these observations and to further evaluate the utility of cytokine and gene expression monitoring as biomarkers of therapeutic response and disease progression in this patient population.

## Figures and Tables

**Table 1:** Baseline characteristics of the study cohort (*n* = 44).

Characteristic	Ph-Neg MPN Patients (*n* = 44)
Number of patients	44
Age (years), median (range)	67 (43–81)
Sex (Male/Female)	27/17
Diagnosis	
Polycythaemia vera	22
Essential thrombocythemia	16
Primary myelofibrosis	6
Driver mutation	
JAK2 V617F	34 (77.3%)
CALR	5 (11.4%)
MPL	2 (4.5%)
Triple-negative	3 (6.8%)
Haemoglobin (g/dL), mean (range)	12.3 (9.6–15.1)
Platelet count (×10^9^/L), mean (range)	426 (130–870)
Leukocyte count (×10^9^/L), mean (range)	9.4 (3.2–17.8)
EBV, HCMV or VZV reactivation	5 (11.4%)
Prior cytoreductive therapy	6 (13.6%)
IFNα treatment duration (months), median (range)	8.2 (1–36)

## Data Availability

The data presented in this study are available on request from the corresponding authors. The data are not publicly available due to ethical and privacy restrictions linked to patient confidentiality.

## Supplementary Materials

The following supporting information can be downloaded at https://www.mdpi.com/article/10.3390/cancers17142273/s1. Figure S1: Immune and clinical features of the Philadelphia-negative MPN cohort at baseline; Table S1: Summary primer sequences, amplicon sizes, and genomic annotations; Table S2: Summary statistics and group comparisons for plasma cytokine levels; Table S3: Gene expression levels in PBMCs; Table S4: Correlations between gene expression and cytokine levels in patients under IFNα treatment.

## Abbreviations

The following abbreviations are used in this manuscript:

AML	Acute myeloid leukaemia
CALR	Calreticulin
cDNA	Complementary DNA
CHIP	Clonal haematopoiesis of Indeterminate Potential
CML	Chronic myeloid leukaemia
CTLA-4	Cytotoxic T Lymphocyte Associated Protein 4
EBV	Epstein-Barr virus
ET	Essential thrombocythemia
HCMV	Cytomegalovirus
HSC	Haematopoietic stem cell
IFNγ	Interferon-gamma
IL-1β	Interleukin-1β
IL-6	Interleukin-6
JAK2	Janus Tyrosine Kinase 2
MCP-1	Monocyte Chemoattractant protein-1
MDS	Myelodysplastic syndromes
MPNs	Myeloproliferative neoplasms
NK	Natural killers
PBMC	Peripheral blood mononuclear cell
PD-1	Programmed Death 1
PD-L1	Programmed Death-Ligand 1
Ph-neg MPNs	Philadelphia-negative myeloproliferative neoplasms
PMF	Primary myelofibrosis
PV	Polycythaemia vera
qPCR	Quantitative real-time PCR
TNF-α	Tumour necrosis factor-alpha
STAT	Signal Transducer and Activator of Transcription
VZV	Varicella Zoster Virus
