# Correction: García-Delgado et al. Translational Insights into Interferon Alpha’s Effects on Immunomolecular Dynamics in Philadelphia-Negative Myeloproliferative Neoplasms. *Cancers* 2025, *17*, 2273

**DOI:** 10.3390/cancers18203345

**Published:** 2026-10-16

**Authors:** Regina García-Delgado, Elena Luque-Lupiáñez, David Mora-Infante, Rodolfo Matías Ortiz-Flores, Borja Cidoncha-Morcillo, Julio Torres-González, Andrés Fontalba-Navas, Alejandro Escamilla-Sánchez

**Affiliations:** 1 UGC Hematología y Hemoterapia, Hospital Universitario Virgen de la Victoria, Servicio Andaluz de Salud, 29010 Malaga, Spain; regina.garcia.delgado.sspa@juntadeandalucia.es (R.G.-D.); elenaluquelupianez@gmail.com (E.L.-L.); borja.cidoncha.sspa@juntadeandalucia.es (B.C.-M.); 2 BE21-Hematología e Inmunoterapia, Instituto de Investigación Biomédica de Málaga y Plataforma en Nanomedicina (IBIMA Plataforma BIONAND), 29071 Malaga, Spain; jtorres.bbm@gmail.com (J.T.-G.); afontalba@uma.es (A.F.-N.); 3 Departamento de Fisiología Humana, Histología Humana, Anatomía Patológica y Educación Físico-Deportiva, Unidad Docente de Histología, Facultad de Medicina, Universidad de Malaga, 29071 Malaga, Spain; davidmorainfante@gmail.com; 4 Departamento de Salud Pública y Psiquiatría, Facultad de Medicina, Universidad de Malaga, 29071 Malaga, Spain; 5 Unidad de Salud Mental, Hospital de Antequera, Área Sanitaria Norte de Malaga, Servicio Andaluz de Salud, 29200 Malaga, Spain

## 1. Error in Figure 3, Supplementary Table S4, and Associated Text

In the original publication [1], reporting inconsistencies were identified in selected Spearman correlation coefficients and their corresponding *p*-values in Section 3.4, Figure 3, and Supplementary Table S4. Following re-verification of the molecular cohort, the complete predefined 7 × 5 gene–cytokine correlation matrix (35 tests) was recalculated de novo from the original patient-level data for all 18 unique patients. Spearman rank correlations were recomputed directly from the continuous source values, with ties handled using standard average ranking. Benjamini–Hochberg false discovery rate (BH-FDR) correction was applied across the complete family of 35 gene–cytokine tests.

The corrected analysis identified four associations that remained statistically significant after BH-FDR correction: STAT1–IL-13 (ρ = 0.819; nominal *p* < 0.001; q = 0.00115), STAT3–IL-6 (ρ = 0.783; nominal *p* < 0.001; q = 0.00214), STAT3–TNFα (ρ = 0.667; nominal *p* = 0.0025; q = 0.02716), and STAT3–IL-10 (ρ = 0.656; nominal *p* = 0.0031; q = 0.02716). MYB–IL-13 (ρ = −0.511; *p* = 0.0302; q = 0.1913) and BCL2–TNFα (ρ = 0.504; *p* = 0.0328; q = 0.1913) were nominal associations only and did not remain significant after multiplicity correction. The previously highlighted STAT3–IL-4 association was not supported (ρ = 0.153; *p* = 0.5452; q = 0.8673).

Figure 3 and Supplementary Table S4 have therefore been replaced with the corrected versions. Statements in Section 3.4 and the Discussion that referred directly to the previous correlation outputs have also been revised to ensure consistency with the verified correlation coefficients, nominal *p*-values, and BH-FDR-adjusted q-values.

**Figure 3:**
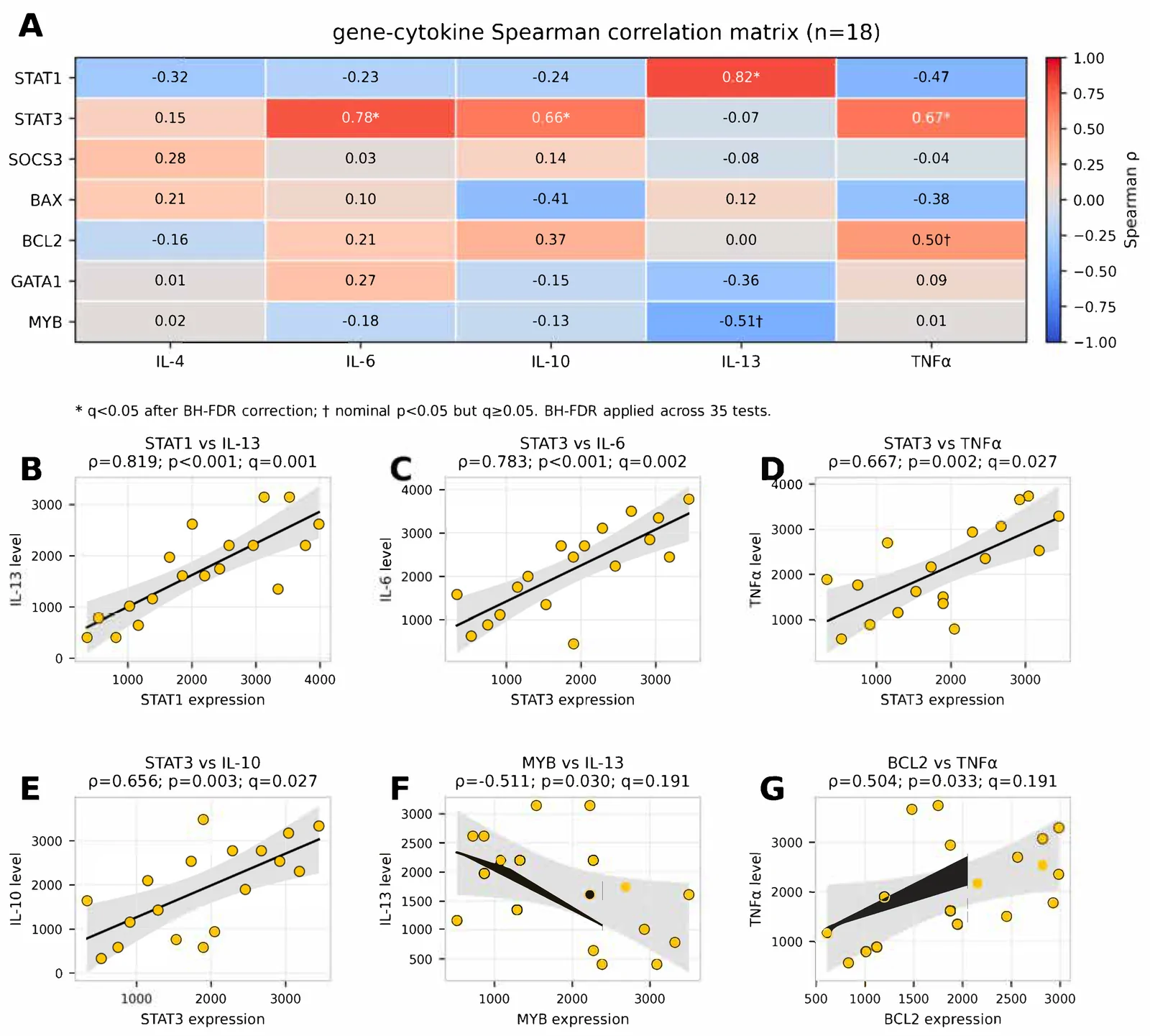
Gene–cytokine correlations in the IFNα-treated molecular cohort. (**A**) shows all predefined 7 × 5 Spearman correlations recalculated from patient-level data (*n* = 18). (**B**–**G**) show FDR-significant associations and nominal-only exploratory associations included in the corrected response. In panels (**B**–**G**), yellow circles represent individual patient observations, the solid black line represents the fitted linear regression line for visualization, and the grey shaded area denotes the 95% confidence interval.

## 2. Revised Figure 3 Legend

Figure 3. Gene–cytokine correlations in the IFNα-treated molecular cohort. (**A**) shows all predefined 7 × 5 Spearman correlations recalculated from patient-level data (*n* = 18). (**B**–**G**) show FDR-significant associations and nominal-only exploratory associations included in the corrected response. In panels (**B**–**G**), yellow circles represent individual patient observations, the solid black line represents the fitted linear regression line for visualization, and the grey shaded area denotes the 95% confidence interval.

## 3. Corrected Supplementary Table S4

Supplementary Table S4 has been replaced by the corrected table entitled “Table S4. Correlations between gene expression and cytokine levels in patients under IFNα treatment.” The revised table reports all 35 predefined gene–cytokine correlations, including Spearman ρ, nominal two-sided *p*-values, BH-FDR-adjusted q-values calculated across the complete 35-test family, and the corrected interpretation of each association.

## 4. Revised Text for Section 3.4

The correlation results in Section 3.4 should read as follows:

“Spearman correlation analysis of the 18-patient molecular cohort identified nominal associations between STAT1 expression and IL-13 (ρ = 0.819; *p* = 0.000033), STAT3 expression and IL-6 (ρ = 0.783; *p* = 0.000122), STAT3 expression and TNFα (ρ = 0.667; *p* = 0.00250), STAT3 expression and IL-10 (ρ = 0.656; *p* = 0.00310), MYB expression and IL-13 (ρ = −0.511; *p* = 0.0302), and BCL2 expression and TNFα (ρ = 0.504; *p* = 0.0328). After Benjamini–Hochberg correction across the complete set of 35 gene–cytokine correlations, STAT1–IL-13 (q = 0.00115), STAT3–IL-6 (q = 0.00214), STAT3–TNFα (q = 0.0272), and STAT3–IL-10 (q = 0.0272) remained statistically significant at FDR < 0.05. MYB–IL-13 and BCL2–TNFα did not remain significant after multiplicity correction (both q = 0.191). These findings are exploratory and require independent validation.”

## 5. Revised Text in the Discussion

To maintain consistency with the corrected Figure 3 and Supplementary Table S4, statements in the Discussion that relied on the previous correlation outputs should be revised as follows:

“The BAX–IL-10 association was inverse but did not reach nominal or FDR-adjusted statistical significance (ρ = −0.412; *p* = 0.0893; q = 0.3905). Accordingly, no mechanistic inference is made from this association, and the BAX/BCL2 expression findings are interpreted independently of this exploratory cytokine correlation [24].”

“MYB–IL-13 showed a nominal inverse association (ρ = −0.511; *p* = 0.0302) that did not remain significant after BH-FDR correction (q = 0.1913). In contrast, STAT1–IL-13 showed a positive association (ρ = 0.819; *p* < 0.001; q = 0.00115) that remained significant after BH-FDR correction. Given the cross-sectional design, these associations are considered exploratory and should not be interpreted as evidence of causal or within-patient temporal relationships [26].”

“Among the predefined gene–cytokine correlations, STAT1–IL-13, STAT3–IL-6, STAT3–TNFα, and STAT3–IL-10 remained statistically significant after BH-FDR correction. MYB–IL-13 and BCL2–TNFα were nominal only, whereas STAT3–IL-4, SOCS3–IL-6, GATA1–IL-10, and BAX–IL-10 were non-significant. The interpretation has therefore been narrowed to avoid mechanistic conclusions based on associations that were not supported after multiplicity correction [29–33].”

These corrections are limited to the presentation and interpretation of the exploratory gene–cytokine correlation analysis. They do not alter the underlying cytokine measurements or RT-qPCR expression data. The corrections narrow the interpretation of specific gene–cytokine associations and preserve the exploratory, hypothesis-generating nature of the molecular substudy.

The authors state that the scientific conclusions are unaffected. This correction was approved by the Academic Editor. The original publication has also been updated.

With this correction the name of the author “Rodolfo Matías Ortiz-Flores” was also updated.
