# Supplementary material for: Translational Insights into Interferon Alpha’s Effects on Immunomolecular Dynamics in Philadelphia-Negative Myeloproliferative Neoplasms
Source: Cancers (Basel). 2025 Jul 8;17(14):2273. doi: 10.3390/cancers17142273 (PMC12293709; doi:10.3390/cancers17142273)
Supplement: Supplementary file 1 [file cancers-17-02273-s001.zip › cancers-3722410-supplementary.pdf]

Table S1. Summary primer sequences, amplicon sizes, and genomic annotations.

Gene Symbol	Full Gene Name	RefSeq ID	Amplicon Size (bp)	Forward Primer (Exon)	Reverse Primer (Exon)	Genomic Location	Forward Primer (5′–3′)	Reverse Primer (5′–3′)
**STAT1**	Signal transducer and activator of transcription 1	NM_007315.3	105	Exon 20	Exon 21	2q32.2–q32.3	CAGCTCAGATGCTGACCAGT	GCTGGTCTTCTCTGCCTTCA
**STAT3**	Signal transducer and activator of transcription 3	NM_139276.2	112	Exon 19	Exon 20	17q21	TAGCCCTGGAGAAGGAGGGA	GTTGCTGGTCTGAGGACGGT
**SOCS1**	Suppressor of cytokine signaling 1	NM_003745.1	112	Exon 2	Exon 2	16p13.13	TTCTTCGCTGTCAGCATCTG	AGGCTGGTGAGGAGAGGAAG
**SOCS3**	Suppressor of cytokine signaling 3	NM_003955.3	68	Exon 2	Exon 2	17q25.3	CCAAGGAGGCTGAGGAGTTT	TTCTTGCCGTAGGCTGTGAC
**CXCL10**	Chemokine (C-X-C motif) ligand 10	NM_001565.3	121	Exon 1	Exon 2	4q21	ATGAGTGCTGCCGCTGAT	CCAGCTTGAGCTTCCTTTTG
**TNFAIP3**	Tumor necrosis factor, alpha-induced protein 3	NM_001270508.1	124	Exon 3	Exon 4	6q23–q25	CCTGAGCCTGTGAGTGTGTG	AGGGTTGATGTTGCTGAGGG
**BAX**	BCL2-associated X protein	NM_004324.3	71	Exon 4	Exon 4	19q13.3–q13.4	CCAAGTGTCTCAGGGAGCTG	TGAGGGTGAGGAGTGAGGAC
**BCL2**	B-cell CLL/lymphoma 2	NM_000633.2	64	Junction 1–2	Exon 2	18q21.3	GGATGCCTTTGTGGAACTGT	CTTGAGCAGAGCAGGGTGAT
**GATA1**	GATA binding protein 1	NM_002049.4	118	Exon 4	Exon 5	Xp11.23	ATGGAGGAACTGGGCAGCTT	GGTGTCCCTGAGTGGTGTCT
**MYB**	v-myb avian myeloblastosis viral oncogene homolog	NM_005375.4	126	Exon 9	Exon 10	6q23.3	CAGTGCCGATCTCTACGTGA	TTGATGGGTTTCTCCTGCGA

Table S2. Summary statistics and group comparisons for plasma cytokine levels.

	IL-1α	IL-1β	IL-6	IL-8	MCP-1	IFNγ	TNFα	IL-4	IL-10	IL-13
**Group I**	8.31 ± 0.16	11.93 ± 1.17	6.93 ± 0.82	2.63 ± 0.75	63.24 ± 11.91	33.50 ± 6.04	8.29 ± 0.40	0.92 ± 0.24	0.75 ± 0.13	0.49 ± 0.14
**Group II**	10.12 ± 1.11	9.10 ± 0.71	7.02 ± 1.44	1.95 ± 0.57	66.96 ± 8.56	17.96 ± 8.81	5.46 ± 1.10	4.41 ± 0.91	15.13 ± 2.50	13.23 ± 2.50
**Group III**	0.66 ± 0.12	1.71 ± 0.40	2.07 ± 0.91	4.20 ± 1.03	92.09 ± 9.70	5.82 ± 1.31	1.63 ± 0.39	3.90 ± 0.49	15.99 ± 1.07	12.45 ± 1.03
**p-value**	1.5e-05	1.8e-05	0.0027	ns	ns	0.00012	5.3e-05	0.0011	0.00016	0.00028

The mean ± SEM of the concentration of each cytokine is shown for Groups I, II, and III. Kruskal-Wallis p values are included in the bottom row. Ns= not statistically significant

Table S3. Gene expression levels in PBMCs.

	CXCL10	TNFAIP3	BAX	BCL2	GATA1	MYB	SOCS1	SOCS3	STAT1	STAT3
**Group I**	187,4 ± 47,4	788,48 ± 46,76	401,85 ± 8,78	506,56 ± 17,61	66,36 ± 21,55	121,74 ± 13,12	209,88 ± 48,57	1356,16 ± 220,95	6912,52 ± 499,76	1813,28 ± 131,41
**Group II**	71,13 ± 7,69	1167,09 ± 251,26	419,15 ± 47,68	262,36 ± 34,14	34,41 ± 6,74	60,87 ± 12,15	55,182 ± 7,4	838,75 ± 45,95	3996,52 ± 644,53	908,91 ± 70,54
**Group III**	148,47 ± 15,91	3399,73 ± 449,56	370,51 ± 16,84	221,85 ± 26,08	14,84 ± 1,83	37,56 ± 4,06	46,35 ± 3,37	597,68 ± 62,06	3810,79 ± 417,26	896,89 ± 52,45
**p-value**	0,0261	0,0022	0,3223	0,0148	0,0521	0,0132	0,0139	0,2688	0,0066	0,7856

The mean ± SEM of each gene (expressed as 2^-ΔCq × 10,000) is shown for Groups I, II, and III. Kruskal–Wallis p values are indicated in the bottom row.

Table S4. Correlations between gene expression and cytokine levels in patients under IFNα treatment.

Gene	Cytokine	Rho (ϱ)	p-value	Significance
STAT1	IL-13	0,99	<0.001	***
MYB	IL-13	-0,68	0.005	**
STAT3	TNFα	0.61	0.008	**
STAT3	IL-4	0,08	0.014	*
BCL2	TNFα	0,03	0.011	*
BAX	IL-10	-0,32	0.053	ns
STAT1	IL-4	-0,16	0.558	ns
SOCS3	IL-6	0,06	0.917	ns
GATA1	IL-10	-0,25	0.951	ns

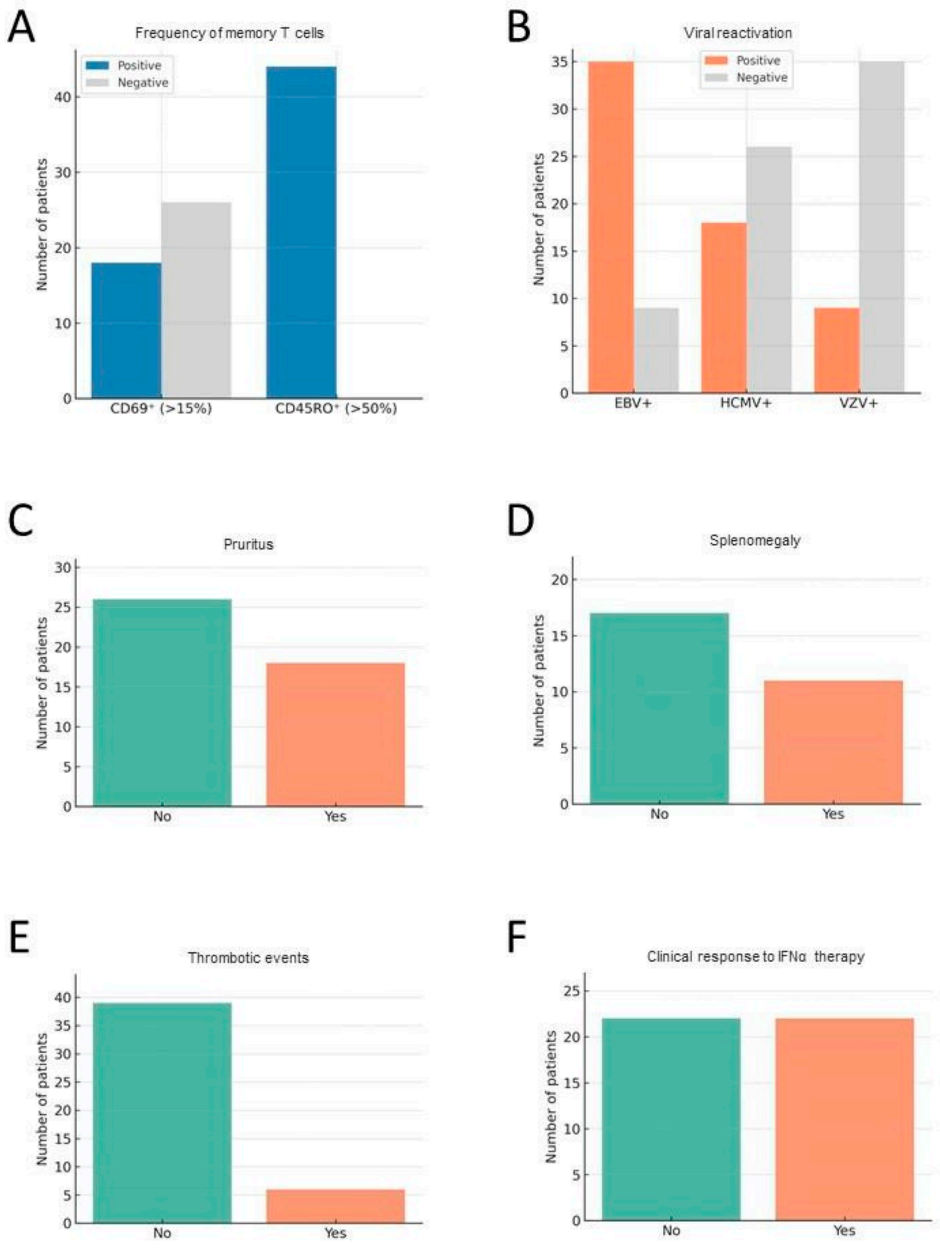

Figure S1. Immune and clinical features of the Philadelphia-negative MPN cohort at baseline. These data demonstrate the clinical and immunological diversity of the cohort, providing a foundation for subsequent correlation analyses. (A) Distribution of $CD69^{+}$ T and $CD45RO^{+}$ memory T cells. (B) Rates of viral reactivation for EBV, HCMV and VZV. (C) Prevalence of pruritus among patients. (D) Proportion of patients with clinically documented splenomegaly. (E) Incidence of current or prior thrombotic events among patients. (F) Overall clinical response to IFNα therapy.
